# Gastric Carcinoma in a Patient with Chronic Lymphocytic Leukemia: Coincidence or Consequence?

**DOI:** 10.7759/cureus.2405

**Published:** 2018-04-02

**Authors:** Mayank Mangal, Sundaramurthi Sudharsanan, TP Elamurugan, Sadasivan Jagdish

**Affiliations:** 1 Surgery, Jawaharlal Institute of Postgraduate Medical Education & Research (JIPMER), Puducherry, IND; 2 Surgery, Jawaharlal Institute of Postgraduate Medical Education and Research (JIPMER), Puducherry, IND

**Keywords:** stomach, adenocarcinoma, cll

## Abstract

Chronic lymphocytic leukemia (CLL) is a neoplasm of mature B-cells of unknown etiology. There is a site-specific increased incidence of second malignancy in patients with CLL. Leukemia and cancer can thus occur in the same patient either simultaneously or sequentially. We present a case of gastric adenocarcinoma in a patient with chronic lymphocytic leukemia.

A 47-year old female presented with a history of abdominal pain for one year, along with nausea and vomiting for two months. On examination, she was pale and had generalized lymphadenopathy. Her abdominal examination revealed vague fullness in the epigastrium, but there was no definite palpable mass. The complete hemogram showed features suggestive of CLL, which was later confirmed by a lymph node biopsy and bone marrow examination. While upper gastrointestinal endoscopy revealed an ulceroproliferative growth in the body of the stomach, its biopsy revealed a well-differentiated adenocarcinoma.

Gastric cancer developing in a patient with CLL may be due to the immunological impairment associated with other etiological factors, such as Helicobacter pylori infection, smoking, etc. The treatment of gastric cancer consists of a gastrectomy with regional lymphadenectomy followed by adjuvant chemotherapy. The co-existence of CLL and carcinoma stomach can pose a challenge in the management of such patients.

## Introduction

Chronic lymphocytic leukemia (CLL) is a neoplasm of mature B cells of unknown etiology. There is a site-specific, increased occurrence of second cancer in patients with CLL, as reported in prior studies. Leukemia and cancer can thus co-exist in the same patient concurrently or successively [1].

## Case presentation

A 47-year-old female presented with a history of upper abdominal pain for six months, which was dull-aching in nature and aggravated after taking food. She gave a history of nausea but there was no vomiting. There was a history of loss of appetite and loss of weight of about 5 kilograms for the past one year. There was no history of jaundice, hematemesis, melena, or any altered bowel habits. On examination, she was thin built, moderately nourished, pale, with generalized lymphadenopathy. Her abdominal examination revealed vague fullness in the epigastrium, but there was no definite palpable mass/organomegaly. The rest of the systemic examination was unremarkable.

The complete hemogram of the patient revealed hemoglobin: 8 g/dl, white blood cell (WBC) count: 59,590 cells/cu mm, absolute lymphocyte count: 48,270/cc, and platelet count: 2,91,000/cc. The peripheral smear showed leucocytosis with numerous mature-appearing lymphoid cells with scanty cytoplasm. The nucleus did not show nucleolus/grooving. Many smudge cells were seen in the background, highly suggestive of a chronic lymphoproliferative disorder, possibly CLL.

**Figure 1:**
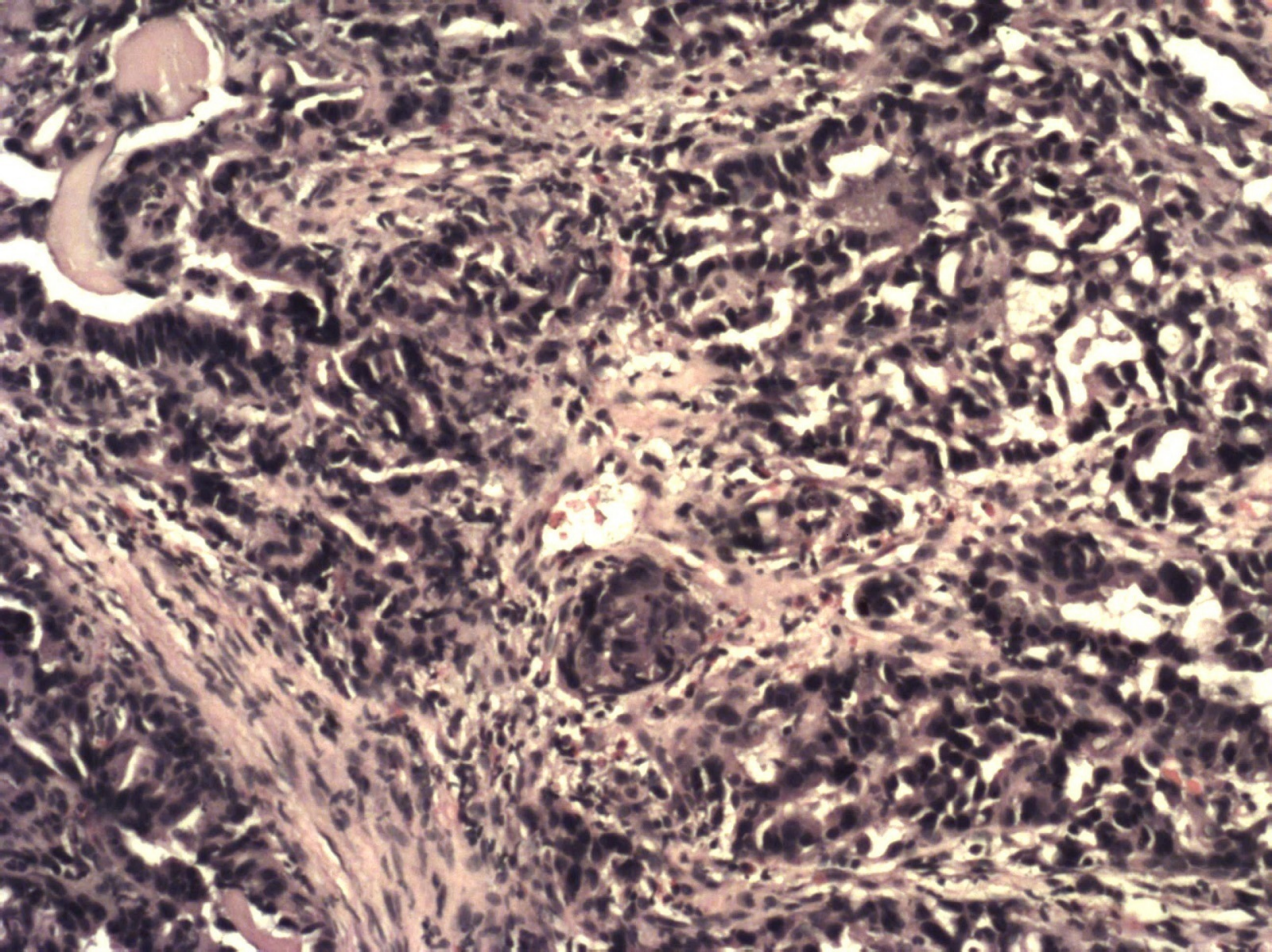
Histopathological examination of the stomach, showing atypical cells consistent with adenocarcinoma

Upper gastrointestinal endoscopy revealed an ulceroproliferative growth in the antropyloric region of the stomach. The biopsy was reported as adenocarcinoma of the stomach-intestinal type, infiltrating the lamina propria (Figure 1).

**Figure 2:**
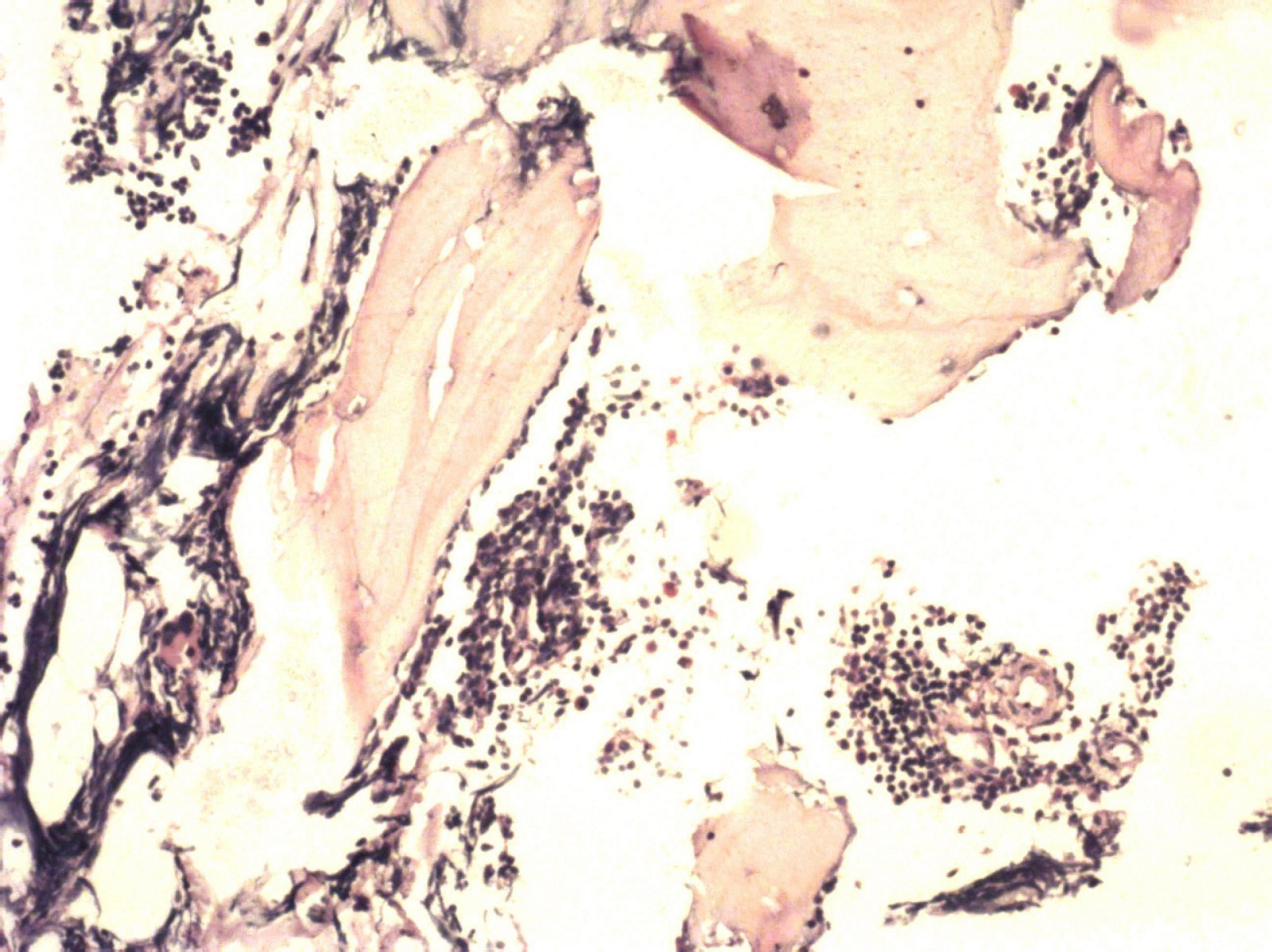
Bone marrow biopsy showing absolute lymphocytosis suggestive of CLL CLL: chronic lymphocytic leukemia

Bone marrow examination revealed hypercellular marrow with absolute lymphocytosis predominantly mature lymphocytes (77%) with many smudge cells and suppressed erythropoiesis and megakaryopoiesis, suggestive of CLL (Figure 2).

**Figure 3:**
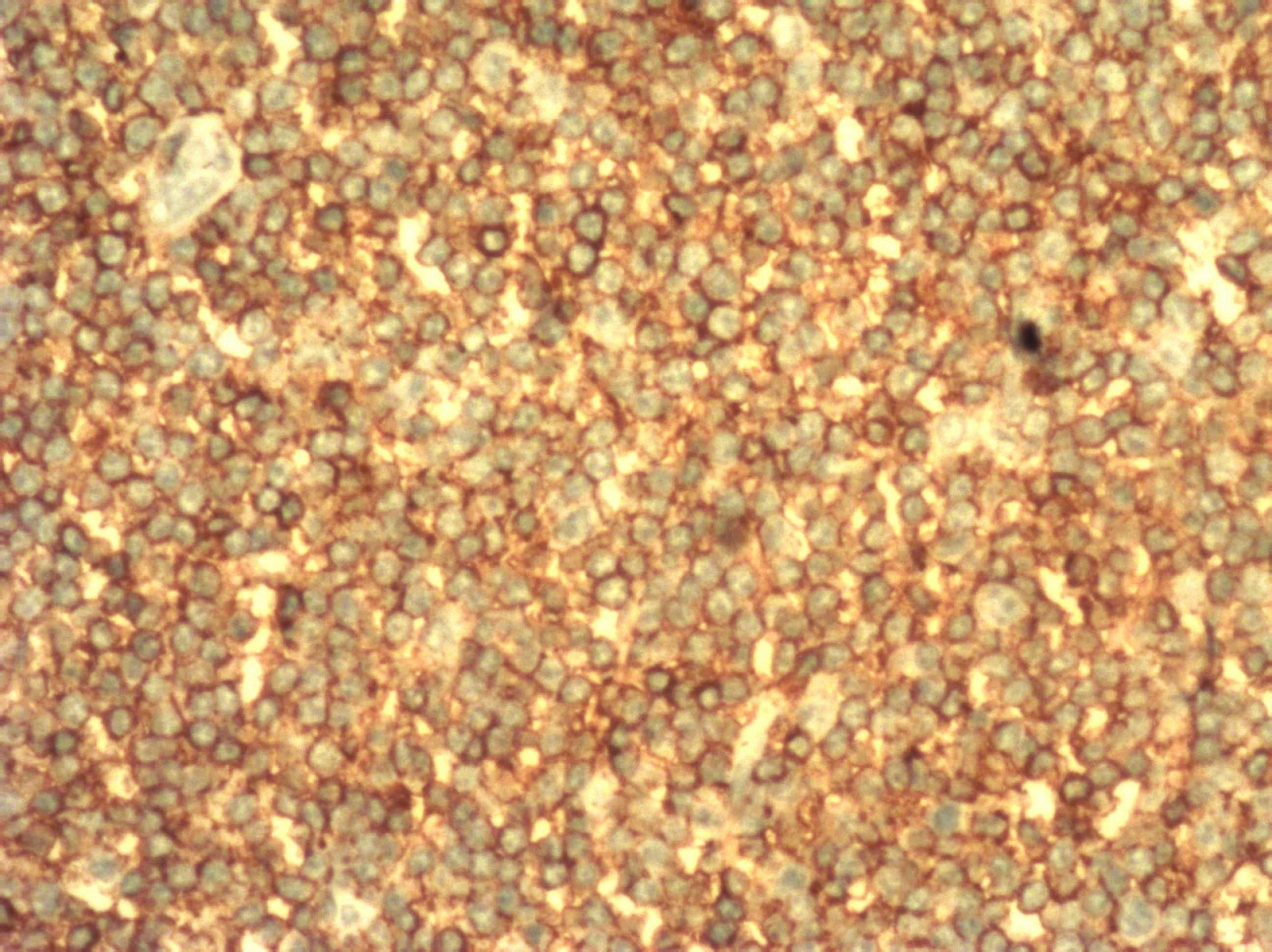
Immunohistochemical sections showing CD5-stained lymphoid cells

**Figure 4:**
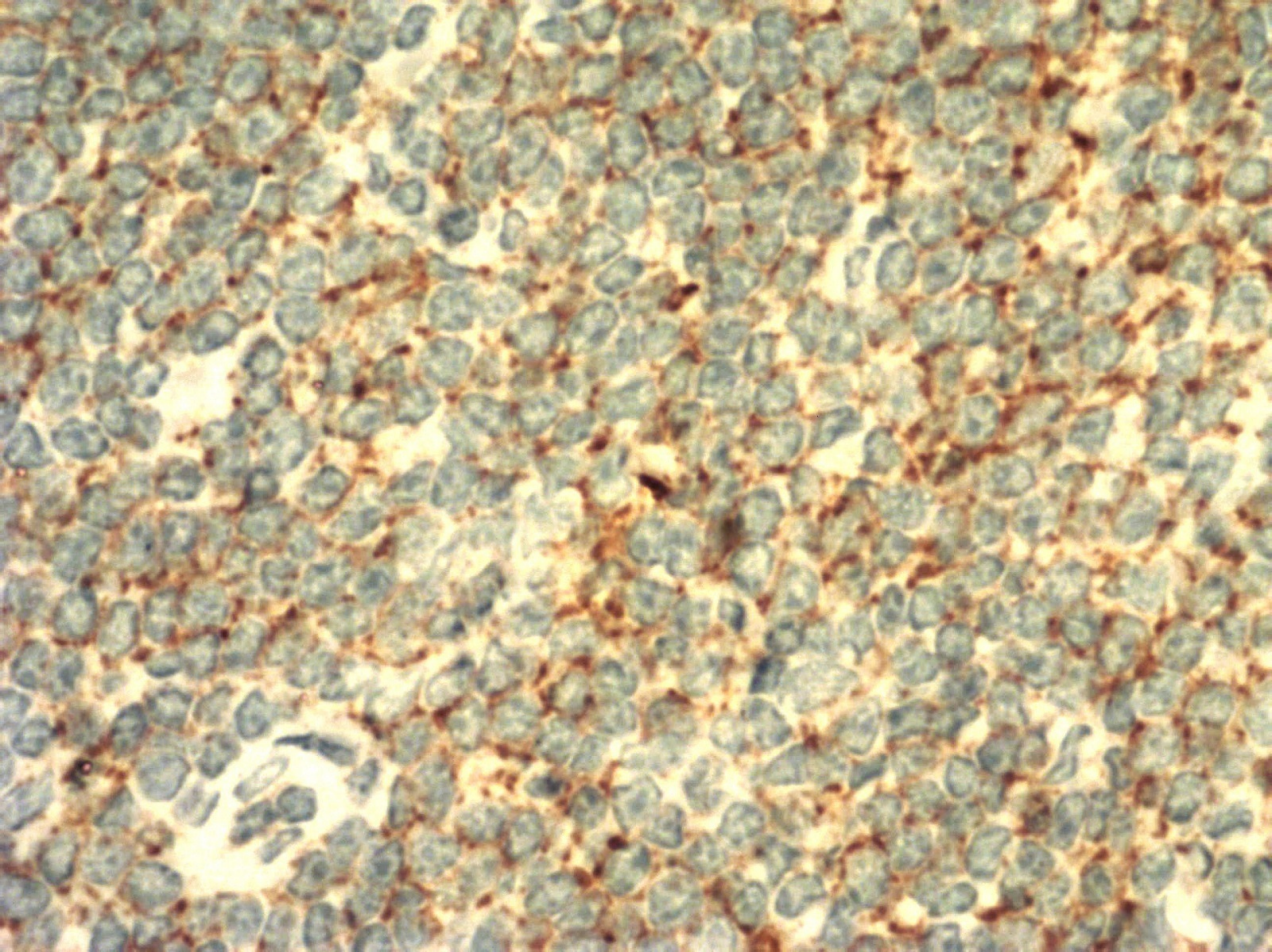
Immunohistochemical sections showing CD20-stained lymphoid cells

**Figure 5:**
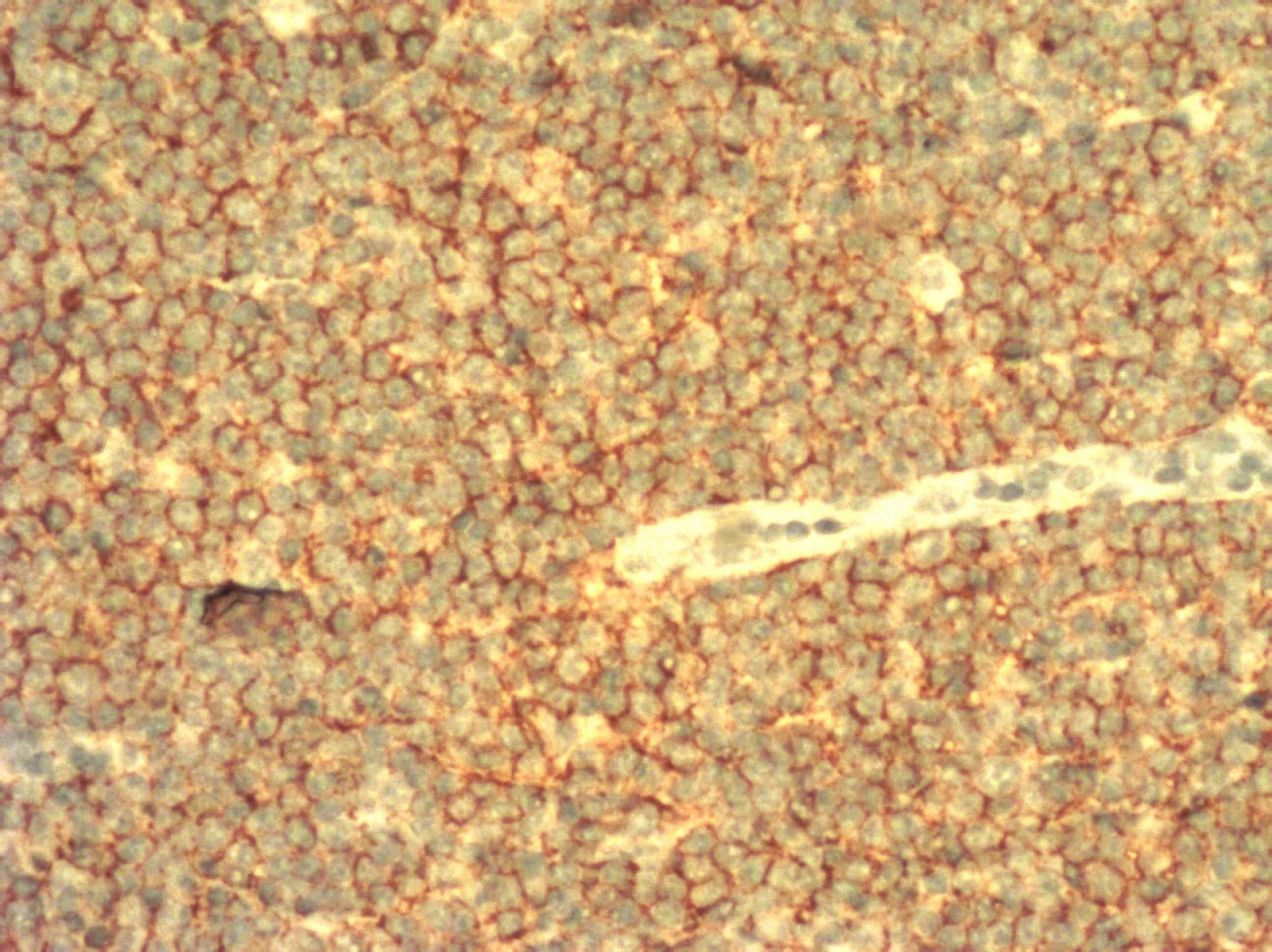
Immunohistochemical sections showing CD23-stained lymphoid cells

**Figure 6:**
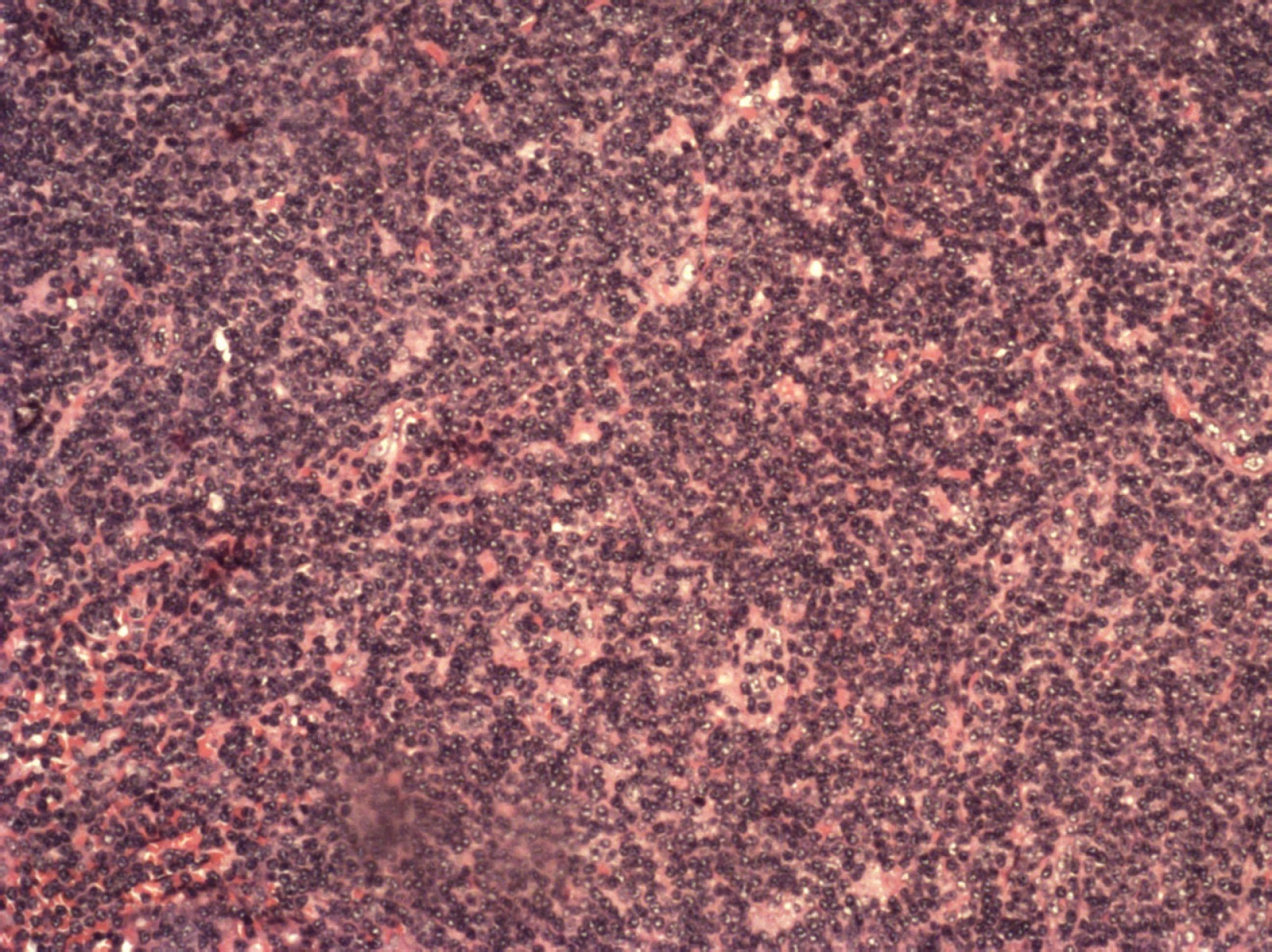
Histopathological examination of lymph node showing infiltration by lymphoid cells

The lymph node biopsy showed diffuse effacement of the architecture with small lymphocytes showing clumped chromatin and scant cytoplasm. CD3-stained reactive T lymphocytes were suggestive of small lymphocytic lymphoma/chronic lymphocytic leukemia (Figures 3-6).

**Figure 7:**
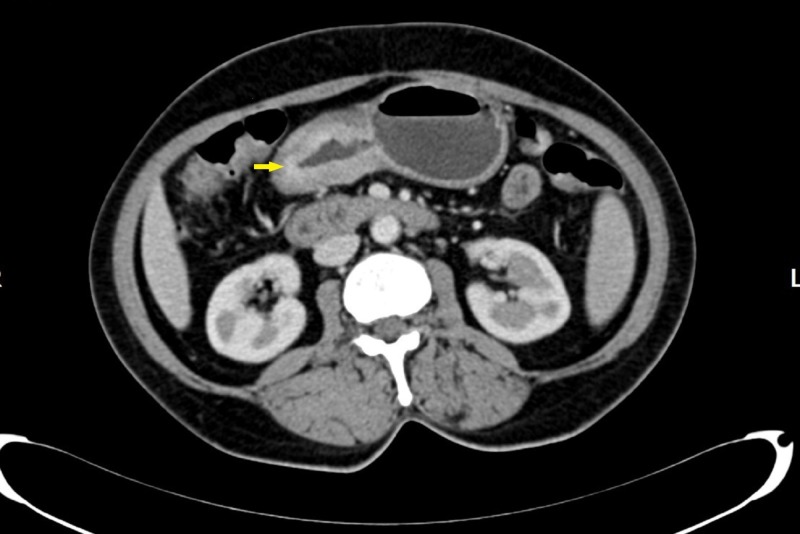
CECT showing enhancing wall thickening of the antropyloric region of the stomach (arrow) CECT: contrast-enhanced computed tomography

Contrast-enhanced computed tomography (CECT) showed an enhancing wall thickening in the antropyloric region for a length of 2 cm with a thickness of 5 cm, anteriorly abutting the posterior surface of the left lobe of the liver with focal loss of the fat plane. A few perigastric nodes and coeliac nodes of 7 mm diameter were present. There was no liver metastasis or free fluid abdomen. Cervical and bilateral axillary lymph nodes were present (Figure 7).

The patient underwent distal radical gastrectomy with D2 lymphadenectomy and is currently planned for adjuvant chemotherapy.

## Discussion

Chronic lymphocytic leukemia is a monoclonal disorder characterized by the accumulation of functionally incompetent lymphocytes. It is a low-grade, indolent, systemic neoplasm of monomorphic, small, round, B-lymphocytes in the peripheral blood, bone marrow, and lymph nodes [2]. It is more common in males with a male to female ratio of 1.7:1. The mean age at presentation is 72 years. It is an acquired disorder characterized by abnormal karyotypes, such as deletions 13q and trisomy 12 [3].

About 25% of the patients are asymptomatic and incidentally diagnosed during the course of investigations for other reasons. The most common presenting symptom is enlarged lymph nodes. Fatigue may be present due to anemia. Petechial hemorrhage may also be present due to thrombocytopenia. In addition, early satiety and abdominal discomfort may present due to splenomegaly.

The infiltration of the gastrointestinal tract by leukemic cells in CLL can manifest as plaques, nodules, multiple leukemic polyposes, and diffuse infiltrations of the mucosal folds. The most common sites involved are the stomach, ileum, and proximal colon, while the duodenum and distal half of the colon are rarely involved [4].

CLL leads to various immunological impairments that can increase the risk of second malignancy. The factors believed to be responsible for the second malignancy are the carcinogenic factors, the initiators and promoters that caused the first cancer, which can be a causative factor for subsequent cancer in other organs. The second factor is the hereditary factor, evident in cases of familial polyposis coli, xeroderma pigmentosum, multiple endocrine adenomatoses, etc. The third factor is the iatrogenic factor, which includes the use of cytotoxic drugs or irradiation, particularly of importance in cancer patients. The fourth factor is an immunosuppressive state caused due to cancer per se [4].

The greatest risk is for skin cancer, especially malignant melanoma and Kaposi sarcoma, other solid tumors, such as renal cell carcinoma, head and neck cancers, lung cancers, and colon cancer, have been reported in association with CLL [1]. The increased risk of stomach cancer among women may be explained by an immune-related predisposition to Helicobacter pylori infection, an important risk factor for gastric carcinoma [1].

Patients with CLL need not be treated with chemotherapy until they become symptomatic or display evidence of rapid progression of disease, as characterized by weight loss of more than 10% over six months, extreme fatigue, fever related to leukemia for longer than two weeks, night sweats for longer than one month, progressive marrow failure (anemia or thrombocytopenia), autoimmune anemia or thrombocytopenia not responding to glucocorticoids, progressive or symptomatic splenomegaly, massive or symptomatic lymphadenopathy, progressive lymphocytosis, as defined by an increase of >50% in two months or a doubling time of less than six months [3].

Chemotherapy is the mainstay for the treatment of CLL. Gastric cancer in CLL can be treated with radical gastrectomy with regional lymphadenectomy.

## Conclusions

CLL and gastric carcinoma can thus co-exist in a single patient, either as a coincidence or a consequence of decreased immunity. Other causative factors for carcinoma stomach in a patient with CLL are yet to be evaluated. The coexistence of a second malignancy in CLL can pose serious challenges in its management. Hence, we recommend routine screening for second malignancy in a patient with CLL.
